# Failure of the glymphatic system by increases of jugular resistance as possible link between asthma and dementia

**DOI:** 10.1093/braincomms/fcae039

**Published:** 2024-02-13

**Authors:** Pasquale Gallina, Francesco Lolli, Duccio Cianti, Francesco Perri, Berardino Porfirio

**Affiliations:** Department of Neurosciences, Psychology, Drug Research and Child Health, University of Florence, 50134 Florence, Italy; Careggi University Hospital, 50134 Florence, Italy; Careggi University Hospital, 50134 Florence, Italy; Department of Clinical and Experimental Biomedical Sciences ‘Mario Serio’, University of Florence, 50134 Florence, Italy; Department of Neurosciences, Psychology, Drug Research and Child Health, University of Florence, 50134 Florence, Italy; Careggi University Hospital, 50134 Florence, Italy; Department of Clinical and Experimental Biomedical Sciences ‘Mario Serio’, University of Florence, 50134 Florence, Italy

Nair *et al*.^1^ provided evidence that relationships between diffusion-weighted magnetic resonance indices of neurodegeneration, CSF biomarkers and decline in cognitive function were consistently stronger in patients with asthma relative to controls. However, the mechanism underlying the association between pulmonary dysfunction and cognitive decline has remained unexplained.

To elaborate on this issue, it would be interesting to have information about a possible quantitative difference in the occurrence of perivascular spaces in the brain of asthmatic patients compared with controls; perivascular spaces are an imaging marker of glymphatic system failure. Perivascular spaces are part of the glymphatic system that is a brain-wide fluid transport pathway involved in the clearance of waste solutes (for the anatomy, physiology, pathology, imaging and clinical relevance of the glymphatic system, see Rasmussen *et al*.^2^ and references within). Subarachnoid CSF enters into arteriolar perivascular spaces via a process known as perivascular space pumping that is driven by arterial wall motion. CSF exchanges with interstitial fluid, providing clearance of interstitial solutes. The fluid then leaves the brain mainly via venous perivascular spaces. CSF transport is facilitated by the expression of aquaporin-4 water channels on the perivascular spaces endfeet of astrocytes. If CSF flow is impaired, perivascular space flushing is less effective^3^ and CSF accumulates and stagnates inside these spaces that become visible at imaging. Derangement of the glymphatic system follows, interstitial waste molecules accumulate and neurodegeneration occurs.

Detection of a higher number of perivascular spaces in the asthmatic patients of the Rosenkranz group^1,4^ compared with controls might support dysfunction of the glymphatic system as responsible for the neurodegeneration in these patients.

We previously hypothesized that a disorder of CSF circulation may involve the upstream failure of extracranial haemodynamics.^5^ Indeed, on the arterial side, impairment of arterial perivascular space flow due to hypertension has been demonstrated,^6^ while on the venous side, impairment of venous perivascular space efflux has been reported in normal pressure hydrocephalus^7^ and presumed as a cause of dementia in patients with severe liver^8^ and pulmonary dysfunction.^9^ In particular, in patients with poor pulmonary function, dementia was hypothesized to be the ultimate phenomenon of a cascade of events involving the reduction of CSF intracranial outflow by increases in jugular resistances, stasis and accumulation of CSF in the interstitium and venous perivascular spaces and glymphatic failure.^9^

Such hypothesis, which would provide an explanation of the findings observed by Rosenkranz’s group,^1,4^ might be assessed by demonstrating the association between dementia and chronic pulmonary heart disease in asthma. Moreover, *in vivo*^10^ and *post-mortem* studies^11^ can investigate findings of glymphatic pathology in cognitively deteriorated asthmatic patients.

Finally, analysis of the duration of venous hypertension, and its burden in these patients, might indicate the threshold of onset of the neurodegenerative process and help individuate the timing for possible CSF shunting.^12,13^ This procedure would aim to ameliorate glymphatic functioning by decongesting interstitium and venous perivascular spaces, thus preventing/limiting neurodegeneration and ultimately dementia.

## Data Availability

Data sharing is not applicable to this article as no new data were created or analysed.
